# Salivary Pro-Inflammatory Markers and Smoking Status Influences the Treatment Effectiveness of Periodontal Disease Patients with Hypertension

**DOI:** 10.3390/ijerph18147364

**Published:** 2021-07-09

**Authors:** Kun-Tsung Lee, Zhu-Ling Guo, Nai-Chia Teng, Kuei-Ling Christine Hsu, I-Hui Chen, Chang-Yu Lee, Hung-Ming Chang, Yung-Kai Huang

**Affiliations:** 1 Department of Oral Hygiene, College of Dental Medicine, Kaohsiung Medical University, Kaohsiung 807, Taiwan; denzelle@kmu.edu.tw; 2 Division of Clinical Dentistry, Kaohsiung Medical University Hospital, Kaohsiung Medical University, Kaohsiung 807, Taiwan; 3 Department of Dentistry, The First Affiliated Hospital of Hainan Medical University, Haikou 571199, China; hy0210026@hainmc.edu.cn; 4 School of Dentistry, Hainan Medical University, Haikou 571199, China; 5 School of Dentistry, College of Oral Medicine, Taipei Medical University, Taipei 110, Taiwan; dianaten@tmu.edu.tw; 6 Department of Dentistry, Taipei Medical University Hospital, Taipei 110, Taiwan; m8504005@tmu.edu.tw; 7 Division of Pediatric Dentistry, Department of Orthodontics and Pediatric Dentistry, University of Maryland School of Dentistry, Baltimore, MD 21201, USA; KHsu@umaryland.edu; 8 Department of Dentistry, Kaohsiung Medical University Hospital, Kaohsiung Medical University, Kaohsiung 807, Taiwan; zakii2710@gmail.com; 9 Department of Anatomy and Cell Biology, School of Medicine, College of Medicine, Taipei Medical University, Taipei 110, Taiwan; taiwanose@tmu.edu.tw

**Keywords:** inflammation and innate immunity, scaling and root planning, non-surgical periodontal therapy, cytokine(s), plaque control

## Abstract

Background: Hypertension and periodontal diseases share several risk factors. Inflammation biomarkers in saliva are related to hypertension and periodontal disease. The aim of this study was to explore the role of the salivary inflammatory biomarkers in the treatment effectiveness of patients with hypertension and periodontal disease. Methods: This observational study enrolled 160 subjects diagnosed with periodontitis, 40 of which had a history of hypertension. All subjects had completed scaling and root planning therapeutic procedures within four weeks. The clinical periodontal parameters (i.e., bleeding on probing, plaque control record (PCR), and probing depth (PD)) were evaluated before and after the treatment. Pro-inflammatory markers were determined using a commercial kit. Results: The recovery rate (PD 4–9 mm) in non-hypertensive subjects was significantly higher than in hypertensive subjects (60.47% vs. 52.60%, respectively; *p* = 0.04). All clinical parameters, excluding PCR, positively correlated with salivary IL-1β at baseline and after completing treatment. Our results showed that increased salivary IL-1β levels were positively associated with decreased PCR (β = −27.65 and *p* = 0.05) and PD recovery rate (β = −17.05 and *p* = 0.02) in hypertensive subjects. Conclusions: The present study sheds important light on the clinical use of salivary pro-inflammatory cytokines as valuable biomarkers for predicting the treatment effectiveness of patients suffering from hypertension and periodontitis.

## 1. Introduction

The periodontium describes tooth-supporting tissues, including gingival tissue, cementum, the periodontal ligament, and alveolar bone. Periodontal diseases are chronic inflammatory diseases, mainly caused by bacteria, that lead to the destruction of the periodontium [1]. The risk of periodontal disease increases with age, poor education, low family income, and high mental stress, and it is closely related to unhealthy lifestyle choices such as smoking, drinking, and betel nut chewing [2,3]. Hypertension and periodontal diseases share several risk factors and by avoiding them, both diseases can be prevented [4]. Due to the high global incidence of both periodontal disease and hypertension, preventive and treatment strategies for these diseases should be consistently emphasized to the public.

Periodontal disease is associated with hypertension and hypertension-related complications, including death from cardiovascular disease or stroke [5]. A nationwide study showed that coronary heart disease related mortality risk was 1.25-fold higher in subjects with periodontitis than those without [6]. This study also highlighted the association between severe periodontitis and intima-media wall thickness (IMT), a subclinical measure of atherosclerosis and severe periodontitis. Furthermore, subjects with severe periodontitis had a 1.31-fold higher probability of having an IMT ≥ 1 mm than those without severe periodontitis [7]. Subjects with periodontal pockets (Community Periodontal Index score of ≥3) have 1.7-fold higher rate of hypertension than those without (Community Periodontal Index score of ≤2) [8]. However, markers for preventing periodontal and chronic vascular diseases or increasing the effectiveness of clinical treatment are yet to be found.

Systemic inflammation positively correlates with cardiovascular disease [9]. Inflammatory cytokine biomarkers, such as interleukin-1 beta (IL-1β), interleukin-6 (IL-6), and tumor necrosis factor alpha (TNF-α) are significantly elevated in patients with cardiovascular and periodontal disease [10]. Other studies have shown similar findings, as an increased expression of inflammatory factors, endothelial dysfunction, high risk of acute thromboembolism, and atherosclerotic plaque instability were all observed in patients suffering from hypertension and periodontal disease [11,12]. It is well known that periodontal pathogens trigger immunologic responses and the production of IL-1β, IL-6, and TNF-α in periodontal tissues [13]. However, the mechanisms underlying the association between local inflammation in periodontal tissues and systemic dissemination via bacteria and inflammatory molecules remain largely unknown and still need to be clarified.

Protein-based salivary profiles have been widely adopted to detect physical condition and identify contributing factors to assess oral disorders, but they have rarely been used to assess the prognosis or treatment of oral diseases [14]. A recent study showed that levels of IL-1β, TNF-α, and other inflammatory cytokines can be used to accurately monitor the progress, treatment effectiveness, and prognosis in periodontal disease [15,16]. Clinical reports have also demonstrated that inflammatory biomarkers could sequence the prognosis of therapy and reflect the status of periodontal disease [17]. However, only a few studies have focused on examining the potential role of inflammatory biomarkers in predicting the treatment effectiveness of periodontal disease along with systemic disorders. Therefore, the current study was designed to explore whether salivary inflammatory cytokines could serve as functional biomarkers in evaluating the treatment effectiveness of patients concurrently suffering from periodontal disease and hypertension.

## 2. Materials and Methods

### 2.1. Subject Recruitment

**Figure 1:**
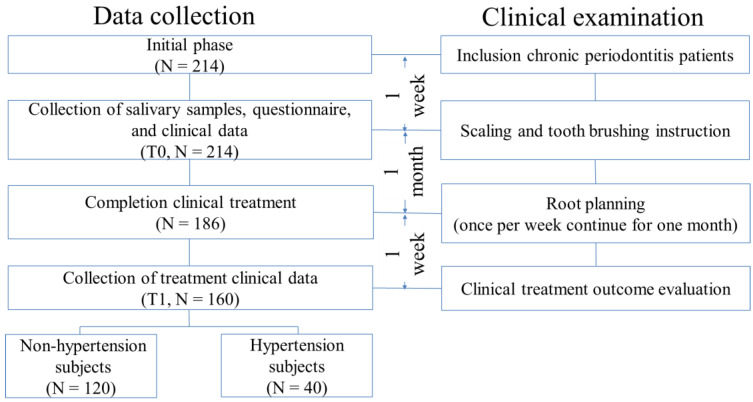
Flowchart of participants’ enrollment.

The patients were enrolled from Department of Periodontology at Medical University Hospital, Northern Taiwan, between July 2013 and April 2016. The patients who had met the following criteria were included in this study: (1) first visit for non-surgical therapy treatment, (2) the number of functional teeth was >15, and (3) the probing depth was ≥5 mm for at least six teeth. Patients who were pregnant or diagnosed with cancer were excluded. The Research Ethics Committee of the Taipei Medical University Hospital Joint Institutional Review Board (Taipei, Taiwan) approved this study, and this study complied with the Declaration of Helsinki. All participants were provided with written informed consent before the questionnaire interview and salivary specimen collection. The flowchart of participant enrolment is show in Figure 1. A total of 214 subjects underwent one-on-one standardized personal interviews by well-trained interviewers for questionnaires, collection of salivary samples, and the collection of clinical data at baseline (T0). After excluding those with incomplete data on clinical indices, 160 subjects (120 non-hypertensive subjects and 40 hypertensive subjects) were recruited at post-treatment follow up (T1). 

Participants who met the inclusion criteria were provided with written informed consent before the questionnaire interview and salivary specimen collection. Questionnaires were carried out through one-on-one interviews by well-trained interviewers. The sociodemographic characteristics (gender and years of schooling) and lifestyle factors (cigarette smoking, alcohol consumption, and betel nut chewing) were included in the structured questionnaire. Questionnaires were administered and saliva specimens were collected before non-surgical therapy treatment. Hypertension was defined as taking hypertension medication and self-reporting. The power analysis for a linear regression was conducted in G-POWER to determine a sufficient sample size using an alpha of 0.05, a power of 0.80, and a small effect size (f^2^ = 0.05) [18]. Based on the aforementioned assumptions, the desired sample size was 159. After excluding those with incomplete hypertension status data, 160 patients (120 non-hypertensive and 40 hypertensive subjects) completed scaling and root planning within four weeks.

### 2.2. Clinical Parameters and Treatment Evaluation

All clinical parametric assessments were performed following published guidelines [19]. Baseline clinical examinations and non-surgical periodontal treatments were carried out by the same periodontist. The plaque control record (PCR) from O’Leary was used to measure plaque [20]. The periodontal probe (Color Coded Michigan Williams Dental Probe) was used to measure bleeding on probing (BOP) and probing depth (PD) at six sites (distobuccal, buccal, mesiobuccal, distolingual, lingual, and mesiolingual) at each tooth. PCR and BOP were expressed as percentages. The average periodontal PD (PD mean in mm) was also calculated. The PD mean was calculated by dividing the sum of all PD sites (mm) by the total number of available sites. The PD 4–6, PD 7–9, and PD 4–9 mm percentages were calculated by dividing the sum of available PD 4–6, PD 7–9, and PD 4–9 mm sites, respectively. The in periodontal clinical parameter differences were calculated as periodontal clinical parameters at baseline (T0) minus periodontal clinical parameters at post-treatment follow-up (T1). The recovery rate of clinical parameters was calculated by dividing the difference periodontal clinical parameters (T0 and T1) by the baseline periodontal clinical parameters (T0).

### 2.3. Specimen Collection and Inflammatory Biomarker Detection

Baseline saliva samples were collected before non-surgical intervention using the Saliva-Check kit (GC Corporation, Tokyo, Japan). The procedure of the storage and treatment of saliva followed that of our previous study [21]. Saliva samples were analyzed for pro-inflammatory biomarkers. The salivary inflammatory biomarkers (IL-1β, IL-6, IL-8, and TNF-α) were determined with an immunoassay using the MILLIPLEX MAP Human Cytokine/Chemokine Magnetic Bead Panel kit (Merck Millipore, Darmstadt, Germany). The coefficients of variance for IL-1β, IL-6, IL-8, and TNF-α were 1.01%, 1.62%, 0.61%, and 0.70%, respectively.

### 2.4. Statistical Analysis

SAS 9.4 (SAS Institute, Cary, NC, USA) was used for all statistical analyses. For categorical variables, the chi-squared test was used to test the association between demographic characteristics and hypertension status. The differences in periodontal clinical parameters (T0 vs. T1) were compared using the Wilcoxon signed-rank test). The Wilcoxon rank-sum test was used to test the differences in recovery rate of clinical parameters and salivary pro-inflammatory biomarkers between non-hypertensive and hypertensive subjects. The strength of the correlation between salivary pro-inflammatory biomarkers and periodontal parameters at baseline or after completing clinical treatment was determined using Spearman’s correlation coefficient.

To explore the association between salivary pro-inflammation biomarkers and hypertension status on treatment effectiveness, multiple general linear regression analyses were used to identify the contributions of demographic characteristics (independent variables), hypertension status, and pro-inflammatory biomarkers to the recovery rate of PD (dependent variables). Independent variables were recorded as follows: gender (female was recorded as (0) and male was recorded as (1)), marital status (single was recorded as (0) and married/separated or divorced was recorded as (1)), smoking status (never having smoked was recorded as (0) and current and former smokers were recorded as (1)), and hypertension status (non-hypertensive was recorded as (0) and hypertensive was recorded as (1)). The levels of pro-inflammatory biomarkers were classified as high or low. The cutoff points distinguishing high and low levels were the median pro-inflammatory biomarker levels of non-hypertensive subjects. The mean levels of IL-1β, IL-6, IL-8, and TNF-α in non-hypertensive subjects were 16.65, 5.45, 451.79, and 4.11 ng/mL, respectively. Pro-inflammatory biomarker expression below and above the mean were recorded as (0) and (1), respectively. The level of significance was set to *p* < 0.05 for all statistical tests.

## 3. Results

The demographic characteristics stratified by the hypertension status of the study participants are described in Table 1. Conversion factors such as age, gender, and smoking status were found to be associated with hypertension status. The average age of the patients was 54.48 years old (standard error: 0.79 years). The percentage of subjects 60 years of age or older was higher in the hypertension group than in the non-hypertension group (52.50 vs. 19.17%, respectively; *p* < 0.001). The hypertension group had a higher percentage of males than the non-hypertension group (60.00 vs. 31.67%, respectively; *p* < 0.01). The percentage of hypertension in married/separated or divorced subjects was higher than in the single subjects (95.00 vs. 5.00%, respectively; *p* = 0.01). The percentage of former smokers was higher in the hypertension group than in the non-hypertension group (27.50 vs. 7.50%, respectively; *p* < 0.01). There were no statistically significant differences in the years of schooling and annual income between the non-hypertension and hypertension groups.

All clinical parameters (Table 2) in the non-hypertension and hypertension groups significantly decreased after treatment. Table 3 shows the clinical parameter recovery rate and salivary inflammatory biomarker levels in subjects based on hypertension disease status. The percentage recovery of PD 7–9 mm in non-hypertensive subjects was 77.78%, which was significantly higher than that observed in hypertensive subjects (77.78 vs. 66.67%, respectively; *p* = 0.03). The percentage recovery of PD 4–9 mm in non-hypertensive subjects was significantly higher than in hypertensive subjects (60.47 vs. 52.60%, respectively; *p* = 0.04). Salivary IL1-β, IL-6, IL-8, and TNF-α levels were also compared between non-hypertensive and hypertensive subjects (Table 3). The levels of all of four salivary inflammatory biomarkers were higher in hypertensive subjects than in non-hypertensive subjects. The levels of IL1-β and IL-8 in hypertensive subjects were borderline significantly higher than those in non-hypertensive subjects (*p* = 0.05 for both).

The salivary inflammatory biomarker levels and periodontal clinical parameters at T0 and T1 are shown in Table 4. All baseline periodontal clinical parameters, excluding PCR (*r* = 0.05; *p* = 0.50), positively correlated with salivary IL1-β levels (*r* = 0.33~0.48; *p* < 0.001) in all subjects. In hypertensive subjects, the correlation coefficients between the salivary IL1-β level and periodontal clinical parameters (*r* = 0.43~0.64) were stronger than those measured in non-hypertensive subjects (*r* = 0.27~0.46). Similar correlation coefficients were observed between salivary IL-8 level and periodontal clinical parameters. After completing treatment, periodontal clinical parameters positively correlated with salivary IL1-β levels (*r* = 0.22~0.42; *p* < 0.001~*p* < 0.01). There were no statistically significant differences in salivary IL-6 and TNF-α in non-hypertensive and hypertensive subjects (Table 3). After the completion of treatment, all periodontal clinical parameters, excluding PCR, positively correlated with salivary IL1-6 (*r* = 0.36~0.47) or TNF-α (*r* = 0.42~0.49).

Table 5 shows that the inflammatory biomarkers levels were not significantly related with the recovery rate of PCR (Models I, V, IX, and XIII) and the recovery rate of PD 4–6 mm (%) (Models III, VI, XI, and XV) in non-hypertensive subjects. In hypertensive subjects, the recovery rate of PCR (%) was significantly lower in those with high salivary IL-1β levels (≥16.65 ng/mL) than the ones with low salivary IL-1β levels (<15.65 ng/mL) (Model II: β = −27.65 and *p* = 0.049). In Models XI and XIV of Table 5, the recovery rate of PCR (%) in hypertensive subjects was significantly lower in subjects with high salivary IL-6 (≥5.45 ng/mL) or TNF-α levels (≥4.11 ng/mL) than those in with low salivary IL-6 (<5.45 ng/mL) or TNF-α levels (<4.11 ng/mL) (β = −27.55, *p* = 0.04 and β = −37.96, *p* = 0.0006, respectively). In hypertensive subjects, the recovery rate of PD 4–6 mm (%) was significantly lower in those with high salivary IL-1β levels (≥16.65 ng/mL) than in those with low salivary IL-1β levels (<15.65 ng/mL) (Model VI: β = −17.05 and *p* = 0.02).

## 4. Discussion

Hypertension has the same risk factors as periodontal disease that is related to many systemic disorders. Our previous study found that there were no significant differences regarding oxidative stress between subjects with and without systemic diseases or medication usage [21]. A meta-analysis study showed that periodontitis is linked to an increased risk of hypertension [22], but little is known about the role of the inflammatory mechanisms shared by both diseases. This study was designed to investigate the potential impacts of hypertension on salivary inflammatory biomarkers and the treatment effectiveness of periodontal disease. When blood pressure is elevated, peripheral resistance increases and the local microcirculation of periodontal tissue is impaired. A high-pressure microenvironment in blood vessels will accelerate the synthesis of inflammatory cytokines and endotoxins induced by periodontal pathogenic bacteria [23,24]. Our results showed that the association between salivary pro-inflammatory biomarkers and clinical periodontal parameters in hypertensive subjects was stronger than that without hypertension. All the pro-inflammatory biomarkers (IL-1β, IL-6, IL-8, and TNF-α) were positively correlated with the clinical parameters at baseline or following completed treatment, except for the PCR in subjects with hypertension. 

IL-1β plays an indispensable role in the inflammatory process and immunity. In the innate immune response, IL-1β induces the synthesis and secretion of other mediators that contribute to inflammatory changes and tissue damage. IL-1β activates the synthesis of nitrous oxide, prostaglandin E2, and platelet-activating factor, which expedite the vascular changes responsible for inflammation and increase blood flow to the injury or infection site. Macrophages, neutrophils, monocytes, keratinocytes, fibroblasts, B cells, osteocytes, and epithelial cells are the primary producers of IL-1β [25]. In endothelial cells, IL-1β stimulates and facilitates neutrophil infiltration into affected tissues by increasing intercellular adhesion molecule 1 expression. Bone resorption is induced by IL-1β synergizing with other proinflammatory cytokines and PGE2. IL-1β also plays a role in adaptive immunity through enhancing the antigen-mediated stimulation of T-cells, stimulating macrophage IL-6 secretion (which in turn activates B-cells), and regulating the development of antigen-presenting cells. IL-1β stimulates the secretion of the chemokine IL-8. [26]. It had been reported that the level of IL-1β in gingival crevicular fluid is increased at sites affected by gingivitis and periodontitis [27]. Moreover, the level of IL-1β correlates well with the clinical severity of periodontal disease [28]. An animal study further supported this viewpoint that a high level of IL-1β will exacerbate inflammation and enhance the resorption of the alveolar bone [29].

Regarding these findings, it has been demonstrated that IL-1β plays a fundamental role in the pathogenesis of periodontal disease [30]. Previous studies have shown that pro-inflammatory biomarkers are significantly elevated in patients with chronic periodontitis [31,32]. The baseline salivary samples were collected to detect the levels of salivary pro-inflammatory biomarkers and predicted the treatment outcomes in patients suffering from periodontal disease both with and without hypertension. This study showed that pro-inflammatory cytokine IL-1β levels are inversely correlated with the recovery rate of periodontitis clinical parameters in hypertensive patients.

TNF-α, an important mediator of *Porphyromonas gingivalis* infection, induces fibroblast apoptosis and osteoclast formation [33]. TNF induces osteoclastogenesis via coupling between the receptor activator of the NF-kB ligand (RANKL) and the TNF type 1 receptor [34]. NF-kB, involved in innate and adaptive immune responses, rapidly activates in response to stimulations such as pathogens, stress signals, and pro-inflammatory cytokines [35]. In chronic periodontitis, the nuclear expression of TNF- α, IL-1b, and IL-8 is significantly up-regulated following NF-kB activation [36]. The level of circulatory TNF-α in non-smoking patients with chronic periodontitis was found to be closely related to the percentage of PD > 7 mm (*r* = −0.41 and *p* = 0.04) at baseline, but the correlation coefficient was reversed to 0.32 (*p* < 0.001) after subgingival scaling [37]. In a cross-sectional study, gingival fluid TNF-α levels were positively associated with blood pressure, which induces a double inflammation effect on patients concurrently suffering from periodontitis and hypertension [38]. It is well known that PD measurement is an essential assessment for periodontal diseases [39]. In the present study, we found that the recovery rate of PD was remarkably lower in hypertensive patients than in non-hypertensive ones. This means that hypertension increases the level of salivary pro-inflammatory biomarkers that subsequently reduce the treatment effectiveness of periodontitis. Based on these findings, our results thus provide a clear and significant correlation between saliva pro-inflammatory biomarkers and the clinical outcomes of periodontitis, particularly in hypertensive patients.

Collecting saliva is a non-invasive and easy sampling method with diagnostic potential in oral diseases. In oral soft tissue, both periodontitis and oral lichen planus are chronic inflammatory diseases. Inflammatory salivary marker levels (such as those of osteopontin and CD44) in patients with oral lichen planus were higher than those in healthy subjects [40]. Saliva contains rich discriminatory protein profiles, and each of them has distinct biological functions [41]. According to their putative applications, biomarkers have been defined as monitoring biomarkers, susceptibility/risk biomarkers, predictive biomarkers, diagnostic biomarkers, and prognostic biomarkers [42]. A diagnostic biomarker of oral cancer development should be incorporated into discovery science and medical product development, and it is needed to ensure sufficient precision and reliability.

In this study, we found statistically significant differences between hypertension and non-hypertension subjects of the following demographic characteristics: age, gender, and marital status. The data from the National Health and Nutrition Examination Survey document indicated an increase in hypertension prevalence with age [43]. A review study pinpointed that the “Vascular Health Triad”—inflammation, oxidative stress, and endothelial dysfunction—was separately implicated in both aging and hypertension [44]. Epidemiological studies have shown that men have higher blood pressure than women before menopause. The gender-associated differences in blood pressure is associated with sex hormones [45]. In a large population-based survey of the United States, the mean hypertension prevalences in never married, married, divorced/separated, and widowed individual were 11.7%, 30.3%, 29.6%, and 57.5%, respectively [46]. The stress of marriage may have detrimental effects and lead to physiological dysregulation [47]. All of the statistically significant different variables in Table 1 had an impact on the association between salivary pro-inflammatory biomarkers and clinical periodontal parameters in hypertensive subjects. Thus, these covariates were adjusted in multiple linear regression models.

Limitations of this study include the misclassification bias of the status of hypertension. The method used for collecting hypertensive subjects in this study was self-reporting, which may have underestimated the number of hypertensive patients due to participants’ unawareness of their own status of hypertension. In order to minimize the misclassification bias, the usage of medication targeted to hypertension was added to the subject’s inclusion list. Under this rigorous selection criterion, the direction of the bias was toward the null and greatly strengthened our results that pointed out the possible role of salivary inflammation status in hypertension subjects in predicting the treatment outcome of periodontitis (Table 3).

We strongly recommend that after the detection of high levels of salivary pro-inflammatory biomarkers, patients might need strict and effective oral hygiene instructions to motivate and promote and behaviors of oral hygiene. Further studies are required to determine the influence of decreased salivary pro-inflammatory biomarkers in periodontal therapy processes in hypertensive patients and to clarify the mechanism by which hypertension, salivary inflammation biomarkers, and oral hygiene affect the quality and effectiveness of periodontal treatment.

## 5. Conclusions

The recovery rate of clinical periodontal parameters was found to be worse in hypertensive subjects when they had high levels of salivary inflammatory biomarkers.

## Figures and Tables

**Table 1:** Demographic characteristics of periodontal disease subjects based on hypertension status.

	Non-Hypertension (*N* = 120)	Hypertension (*N* = 40)	χ^2^ *p* Value
Age, *n* (%)			<0.001
<50 years	38 (31.67)	4 (10.00)	
50–55 years	26 (21.67)	11 (27.50)	
55–50 years	33 (27.50)	4 (10.00)	
>60	23 (19.17)	21 (52.50)	
Gender, *n* (%)			<0.01
Female	82 (68.33)	16 (40.00)	
Male	38 (31.67)	24 (60.00)	
Marital status, N (%)			0.01
Single	28 (23.33)	2 (5.00)	
Married/separated or divorced	92 (76.67)	38 (95.00)	
Years of schooling, N (%)			0.26
≤9 years	10 (8.33)	5 (12.50)	
10~12 years	27 (22.50)	13 (32.50)	
≥13 years	83 (69.17)	22 (55.00)	
Annual income (NTD), N (%)			0.37
<200,000	31 (25.83)	11 (27.50)	
200,000~500,000	16 (13.33)	3 (7.50)	
500,000~1,000,000	25 (20.83)	4 (10.00)	
>1,000,000	19 (15.83)	9 (22.50)	
Missing	29 (24.17)	13 (32.50)	
Smoking, *n* (%)			<0.01
Non-smokers	101 (84.17)	25 (52.50)	
Former smokers	9 (7.50)	11 (27.50)	
Smokers	10 (8.33)	4 (10.00)	

**Table 2:** Clinical parameters at baseline and after periodontal treatment based on hypertension status.

Clinical Parameters	Non-HT (N = 120)		Hypertension (N = 40)	
	Median	Q1–Q3	Median	Q1–Q3
Plaque index (%)				
At the baseline	56.7	44.1–67.97	60.91	44.46–77.58
After completing treatment	33.95	25.6–44.1	37.5	24.66–49.15
*p*-value ^a^	<0.001		<0.001	
Bleeding on probing (%)				
At the baseline	41.51	30.65–54.91	45.1	27.24–67.15
After completing treatment	18.47	13.51–30.4	23.47	15.29–31.07
*p*-value ^a^	<0.001		<0.001	
PD mean (mm)				
At the baseline	3.36	3.09–3.83	3.35	3.17–3.83
After completing treatment	2.73	2.56–2.99	2.86	2.69–3.11
*p*-value ^a^	<0.001		<0.001	
PD 4–6 mm (%)				
At the baseline	23.86	16.04–31.65	22.88	17.77–32.46
After completing treatment	9.70	6.25–14.40	12.6	6.72–16.98
*p*-value ^a^	<0.001		<0.001	
PD 7–9 mm (%)				
At the baseline	3.35	1.75–9.06	3.57	2.08–8.43
After completing treatment	0.64	0–2.04	1.23	0.29–2.08
*p*-value ^a^	<0.001		<0.001	
PD 4–9 mm (%)				
At the baseline	27.97	19.09–38.96	24.68	22.56–40.43
After completing treatment	10.60	6.79–16.67	13.39	7.14–20.03
*p*-value ^a^	<0.001		<0.001	

^a^ Wilcoxon signed-rank test.

**Table 3:** Clinical parameters for recovery rate and salivary inflammatory biomarker levels in subjects based on by hypertension status.

	Non-HT (N = 120)		Hypertension (N = 40)		*p*-Value ^b^
	Median	Q1–Q3	Median	Q1–Q3	
Recovery rate of clinical parameters (%)					
Plaque index (%)	39.77	13.11–54.81	36.79	13.81–50.19	0.84
Bleeding on probing (%)	53.70	27.02–69.01	52.76	27.66–66.23	0.78
Mean of probing depth (mm)	0.64	0.45–0.92	0.63	0.39–0.77	0.39
PD 4–6 mm percentage (%)	55.44	40.63–68.03	45.96	29.09–68.39	0.15
PD 7–9 mm percentage (%)	77.78	51.32–100.00	66.67	25.00–81.53	0.03
PD 4–9 mm percentage (%)	60.47	49.04–70.43	52.60	40.59–69.05	0.04
Salivary inflammatory biomarker levels					
IL1-β(ng/mL)	16.65	4.3–47.8	22.27	6.28–124.11	0.05
IL-6 (ng/mL)	5.45	2.27–9.75	5.16	3.02–12.74	0.46
IL-8 (ng/mL)	415.79	228.23–651.11	544.16	281.75–864.11	0.05
TNF-α (ng/mL)	4.11	1.02–8.49	5.51	1.03–10.77	0.50

^b^ Wilcoxon rank-sum test.

**Table 4:** The spearman’s correlation coefficient between salivary inflammatory biomarker levels and periodontal clinical parameters based on hypertension status.

	Non-HT (N = 120)				HT (N = 40)			
	IL1-β	IL-6	IL-8	TNF-α	IL1-β	IL-6	IL-8	TNF-α
Periodontal clinical parameters at baseline.								
PCR (%)	0.09	0.03	0.12	−0.17	−0.08	0.04	0.22	−0.15
BOP (%)	0.42 ***	0.20 *	0.26 **	0.006	0.55 ***	0.18	0.44 **	0.33 *
PD mean (mm)	0.42 ***	0.11	0.25 **	−0.05	0.57 ***	0.57 ***	0.37 *	0.45 **
PD 4–6mm (%)	0.27 **	0.10	0.18	−0.02	0.43 **	0.37 *	0.43 **	0.35 *
PD 7–9mm (%)	0.46 ***	0.07	0.21 *	−0.08	0.64 ***	0.58 ***	0.26	0.48 **
PD 4–9mm (%)	0.37 ***	0.11	0.22 *	−0.05	0.52 ***	0.48 **	0.38 *	0.45 **
Periodontal clinical parameters after completing treatment.								
PCR (%)	0.19 *	0.07	0.04	−0.02	0.25	0.22	0.22	0.23
BOP (%)	0.22 *	0.11	0.06	−0.03	0.59 ***	0.36 *	0.48 **	0.49 **
PD mean (mm)	0.33 **	0.02	0.12	−0.01	0.61 ***	0.47 **	0.37 *	0.46 **
PD 4–6mm (%)	0.27 **	0.02	0.14	−0.03	0.52 ***	0.47 **	0.32 *	0.42 **
PD 7–9mm (%)	0.33 ***	0.02	0.20 *	−0.06	0.56 ***	0.47 **	0.05	0.49 **
PD 4–9mm (%)	0.30 ***	0.01	0.17	−0.05	0.57 ***	0.47 **	0.30	0.45 **

* *p* < 0.05; ** *p* < 0.01; *** *p* < 0.001.

**Table 5:** Multiple linear regression of periodontal clinical parameter recovery rate in salivary inflammatory biomarkers and conventional risk factors of periodontal disease based on hypertension status.

	Dependent Variable	Recovery Rate of PI (%)		Recovery Rate of PD 4–6 mm (%)	
		Non HT	HT	Non HT	HT
Independent Variable		β (SE)	β (SE)	β (SE)	β (SE)
		Model I	Model II	Model III	Model IV
Salivary IL-1β level		−7.40 (8.37)	−27.65 (13.58) *	−0.19 (3.84)	−17.05 (7.15) *
Smoking status		−3.54 (13.10)	2.76 (14.87)	−4.75 (6.01)	−26.8 (7.83) **
Age		4.97 (3.88)	9.37 (6.26)	4.89 (1.78) *	1.20 (3.30)
Gender		3.72 (10.29)	−21.17 (14.67)	−0.75 (4.73)	7.15 (7.73)
Marital status		−6.55 (10.22)	−24.08 (31.11)	−2.98 (4.69)	19.23 (16.39)
		Model V	Model VI	Model VII	Model VIII
Salivary IL-6 level		0.81 (8.58)	−27.55 (12.90) *	2.30 (3.92)	−6.58 (7.29)
Smoking status		−4.32 (13.14)	7.08 (14.69)	−4.99 (6.01)	−24.44 (8.31) *
Age		5.23 (3.88)	9.83 (6.22)	4.9 (1.77) *	1.38 (3.52)
Gender		3.55 (10.45)	−11.31 (14.48)	−1.18 (4.78)	11.6 (8.19)
Marital status		−6.42 (10.27)	−28.95 (30.91)	−3.15 (4.69)	16.82 (17.48)
		Model IX	Model X	Model XI	Model XII
Salivary IL-8 level		10.67 (8.45)	−14.09 (15.20)	−5.1 (3.87)	−10.98 (8.05)
Smoking status		−4.52 (13.03)	9.63 (15.86)	−4.63 (5.96)	−22.01 (8.4) *
Age		4.64 (3.88)	9.32 (6.55)	5.18 (1.78) **	1.14 (3.47)
Gender		2.63 (10.29)	−20.37 (15.92)	−0.23 (4.71)	6.88 (8.44)
Marital status		−7.35 (10.21)	−23.51 (32.76)	−2.5 (4.67)	20.21 (17.36)
		Model XIII	Model XIV	Model XV	Model XVI
Salivary TNF-α level		−5.00 (8.38)	−37.96 (13.04) **	−3.99 (3.82)	−12.74 (7.52)
Smoking status		−4.85 (13.14)	8.25 (14.01)	−5.25 (5.99)	−23.96 (8.08) *
Age		5.09 (3.88)	9.48 (5.93)	4.79 (1.77) *	1.29 (3.42)
Gender		3.94 (10.32)	−27.22 (14.23)	−0.55 (4.71)	6.68 (8.21)
Marital status		−6.51 (10.24)	−42.81 (29.91)	−3.1 (4.67)	12.01 (17.25)

* *p* < 0.05; ** *p* < 0.01. SE: standard error. HT: hypertension.

## Data Availability

Data sharing not applicable. No new data were created or analyzed in this study. Data sharing is not applicable to this article.

## References

[1] Sanz M., Beighton D., Curtis M.A., Cury J.A., Dige I., Dommisch H., Ellwood R., Giacaman R.A., Herrera D., Herzberg M.C. (2017). Role of microbial biofilms in the maintenance of oral health and in the development of dental caries and periodontal diseases. Consensus report of group 1 of the Joint EFP/ORCA workshop on the boundaries between caries and periodontal disease. J. Clin. Periodontol..

[2] Barros S.P., Offenbacher S. (2014). Modifiable risk factors in periodontal disease: Epigenetic regulation of gene expression in the inflammatory response. Periodontology 2000.

[3] Genco R.J., Borgnakke W.S. (2013). Risk factors for periodontal disease. Periodontology 2000.

[4] Joffres M., Falaschetti E., Gillespie C., Robitaille C., Loustalot F., Poulter N., McAlister F.A., Johansen H., Baclic O., Campbell N. (2013). Hypertension prevalence, awareness, treatment and control in national surveys from England, the USA and Canada, and correlation with stroke and ischaemic heart disease mortality: A cross-sectional study. BMJ Open.

[5] Beck J.D., Papapanou P.N., Philips K.H., Offenbacher S. (2019). Periodontal Medicine: 100 Years of Progress. J. Dent. Res..

[6] DeStefano F., Anda R.F., Kahn H.S., Williamson D.F., Russell C.M. (1993). Dental disease and risk of coronary heart disease and mortality. BMJ.

[7] Beck J.D., Elter J.R., Heiss G., Couper D., Mauriello S.M., Offenbacher S. (2001). Relationship of periodontal disease to carotid artery intima-media wall thickness: The atherosclerosis risk in communities (ARIC) study. Arterioscler. Thromb. Vasc. Biol..

[8] Morita T., Yamazaki Y., Mita A., Takada K., Seto M., Nishinoue N., Sasaki Y., Motohashi M., Maeno M. (2010). A Cohort Study on the Association Between Periodontal Disease and the Development of Metabolic Syndrome. J. Periodontol..

[9] Carrizales-Sepulveda E.F., Ordaz-Farias A., Vera-Pineda R., Flores-Ramirez R. (2018). Periodontal Disease, Systemic Inflammation and the Risk of Cardiovascular Disease. Heart Lung Circ..

[10] Chistiakov D.A., Orekhov A.N., Bobryshev Y.V. (2016). Links between atherosclerotic and periodontal disease. Exp. Mol. Pathol..

[11] Higashi Y., Goto C., Jitsuiki D., Umemura T., Nishioka K., Hidaka T., Takemoto H., Nakamura S., Soga J., Chayama K. (2008). Periodontal Infection Is Associated With Endothelial Dysfunction in Healthy Subjects and Hypertensive Patients. Hypertension.

[12] Schenkein H.A., Loos B.G. (2013). Inflammatory mechanisms linking periodontal diseases to cardiovascular diseases. J. Clin. Periodontol..

[13] Nguyen C.M., Kim J.W.M., Quan V.H., Nguyen B.H., Tran S.D. (2015). Periodontal associations in cardiovascular diseases: The latest evidence and understanding. J. Oral Biol. Craniofacial Res..

[14] Lorenzo-Pouso A.I., Perez-Sayans M., Bravo S.B., Lopez-Jornet P., Garcia-Vence M., Alonso-Sampedro M., Carballo J., Garcia-Garcia A. (2018). Protein—Based Salivary Profiles as Novel Biomarkers for Oral Diseases. Dis. Markers.

[15] de Lima C.L., Acevedo A.C., Grisi D.C., Taba M., Guerra E., De Luca Canto G. (2016). Host—Derived salivary biomarkers in diagnosing periodontal disease: Systematic review and meta-analysis. J. Clin. Periodontol..

[16] Jaedicke K.M., Preshaw P.M., Taylor J.J. (2016). Salivary cytokines as biomarkers of periodontal diseases. Periodontology 2000.

[17] Lee C.H., Chen Y.W., Tu Y.K., Wu Y.C., Chang P.C. (2018). The potential of salivary biomarkers for predicting the sensitivity and monitoring the response to nonsurgical periodontal therapy: A preliminary assessment. J. Periodontal. Res..

[18] Faul F., Erdfelder E., Lang A.G., Buchner A. (2007). G* Power 3: A flexible statistical power analysis program for the social, behavioral, and biomedical sciences. Behav. Res. Methods.

[19] Hefti A.F., Preshaw P.M. (2012). Examiner alignment and assessment in clinical periodontal research. Periodontology 2000.

[20] O’Leary T.J., Drake R.B., Naylor J.E. (1972). The plaque control record. J. Periodontol..

[21] Chang C.H., Han M.L., Teng N.C., Lee C.Y., Huang W.T., Lin C.T., Huang Y.K. (2018). Cigarette Smoking Aggravates the Activity of Periodontal Disease by Disrupting Redox Homeostasis—An Observational Study. Sci Rep..

[22] Muñoz Aguilera E., Suvan J., Buti J., Czesnikiewicz-Guzik M., Barbosa Ribeiro A., Orlandi M., Guzik T.J., Hingorani A.D., Nart J., D’Aiuto F. (2019). Periodontitis is associated with hypertension: A systematic review and meta-analysis. Cardiovasc. Res..

[23] Chukkapalli S.S., Easwaran M., Rivera-Kweh M.F., Velsko I.M., Ambadapadi S., Dai J., Larjava H., Lucas A.R., Kesavalu L. (2017). Sequential colonization of periodontal pathogens in induction of periodontal disease and atherosclerosis in LDLRnull mice. Pathog. Dis..

[24] Hamilton J.A., Hasturk H., Kantarci A., Serhan C.N., Van Dyke T. (2017). Atherosclerosis, Periodontal Disease, and Treatment with Resolvins. Curr. Atheroscler. Rep..

[25] Delaleu N., Bickel M. (2004). Interleukin-1beta and interleukin-18: Regulation and activity in local inflammation. Periodontology 2000.

[26] Ben-Sasson S.Z., Hu-Li J., Quiel J., Cauchetaux S., Ratner M., Shapira I., Dinarello C.A., Paul W.E. (2009). IL-1 acts directly on CD4 T cells to enhance their antigen-driven expansion and differentiation. Proc. Natl. Acad. Sci. USA.

[27] Offenbacher S., Barros S., Mendoza L., Mauriello S., Preisser J., Moss K., De Jager M., Aspiras M. (2010). Changes in gingival crevicular fluid inflammatory mediator levels during the induction and resolution of experimental gingivitis in humans. J. Clin. Periodontol..

[28] Stashenko P., Fujiyoshi P., Obernesser M.S., Prostak L., Haffajee A.D., Socransky S.S. (1991). Levels of interleukin 1β in tissue from sites of active periodontal disease. J. Clin. Periodontol..

[29] Koide M., Suda S., Saitoh S., Ofuji Y., Suzuki T., Yoshie H., Takai M., Ono Y., Taniguchi Y., Hara K. (1995). In vivo administration of IL-1β accelerates silk ligature-induced alveolar bone resorption in rats. J. Oral Pathol. Med..

[30] Kornman K.S., Page R.C., Tonetti M.S. (1997). The host response to the microbial challenge in periodontitis: Assembling the players. Periodontology 2000.

[31] Passoja A., Puijola I., Knuuttila M., Niemela O., Karttunen R., Raunio T., Tervonen T. (2010). Serum levels of interleukin-10 and tumour necrosis factor-alpha in chronic periodontitis. J. Clin. Periodontol..

[32] Ertugrul A.S., Sahin H., Dikilitas A., Alpaslan N., Bozoglan A. (2013). Comparison of CCL28, interleukin-8, interleukin-1beta and tumor necrosis factor-alpha in subjects with gingivitis, chronic periodontitis and generalized aggressive periodontitis. J. Periodontal Res..

[33] Graves D.T., Oskoui M., Volejnikova S., Naguib G., Cai S., Desta T., Kakouras A., Jiang Y. (2001). Tumor necrosis factor modulates fibroblast apoptosis, PMN recruitment, and osteoclast formation in response to P. gingivalis infection. J. Dent. Res..

[34] Zhang Y.H., Heulsmann A., Tondravi M.M., Mukherjee A., Abu-Amer Y. (2001). Tumor necrosis factor-alpha (TNF) stimulates RANKL-induced osteoclastogenesis via coupling of TNF type 1 receptor and RANK signaling pathways. J. Biol. Chem..

[35] Li Q., Verma I.M. (2002). NF-kappaB regulation in the immune system. Nat. Rev. Immunol..

[36] Milward M.R., Chapple I.L.C., Wright H.J., Millard J.L., Matthews J.B., Cooper P.R. (2007). Differential activation of NF-kappa B and gene expression in oral epithelial cells by periodontal pathogens. Clin. Exp. Immunol..

[37] Ide M., Jagdev D., Coward P.Y., Crook M., Barclay G.R., Wilson R.F. (2004). The Short-Term Effects of Treatment of Chronic Periodontitis on Circulating Levels of Endotoxin, C-Reactive Protein, Tumor Necrosis Factor-α, and Interleukin-6. J. Periodontol..

[38] Khocht A., Rogers T., Janal M.N., Brown M. (2017). Gingival Fluid Inflammatory Biomarkers and Hypertension in African Americans. JDR Clin. Trans. Res..

[39] Greenstein G. (1997). Contemporary Interpretation of Probing Depth Assessments: Diagnostic and Therapeutic Implications. A Literature Review. J. Periodontol..

[40] Santarelli A., Mascitti M., Rubini C., Bambini F., Zizzi A., Offidani A., Ganzetti G., Laino L., Cicciù M., Muzio L.L. (2015). Active inflammatory biomarkers in oral lichen planus. Int. J. Immunopathol. Pharmacol..

[41] Yoshizawa J.M., Schafer C.A., Schafer J.J., Farrell J.J., Paster B.J., Wong D.T.W. (2013). Salivary Biomarkers: Toward Future Clinical and Diagnostic Utilities. Clin. Microbiol. Rev..

[42] Califf R.M. (2018). Biomarker definitions and their applications. Exp. Biol. Med..

[43] Mozaffarian D., Benjamin E.J., Go A.S., Arnett D.K., Blaha M.J., Cushman M., De Ferranti S., Després J.-P., Fullerton H.J., Howard V.J. (2015). Heart Disease and Stroke Statistics—2015 Update. Circulation.

[44] Buford T.W. (2016). Hypertension and aging. Ageing Res. Rev..

[45] Dubey R. (2002). Sex hormones and hypertension. Cardiovasc. Res..

[46] Stimpson J.P., Wilson F.A. (2009). Cholesterol screening by marital status and sex in the United States. Prev. Chronic Dis..

[47] Cornwell E.Y., Waite L.J. (2012). Social Network Resources and Management of Hypertension. J. Health Soc. Behav..

